# Advances in the Application of Three‐Dimensional Reconstruction in Thoracic Surgery: A Comprehensive Review

**DOI:** 10.1111/1759-7714.70159

**Published:** 2025-08-29

**Authors:** Guihu Lin, Ruzhen Li, Xiao Li, Dawei Wang, Xiuyuan Chen

**Affiliations:** ^1^ Department of Thoracic Surgery Peking University People's Hospital Beijing China; ^2^ Thoracic Oncology Institute Peking University People's Hospital Beijing China; ^3^ Research Unit of Intelligence Diagnosis and Treatment in Early Non‐Small Cell Lung Cancer, Chinese Academy of Medical Sciences Peking University People's Hospital Beijing China; ^4^ Institute of Advanced Clinical Medicine Peking University Beijing China; ^5^ Beijing Key Laboratory of Innovative Application of Big Data in Lung Cancer Peking University People's Hospital Beijing China; ^6^ Institute of Advanced Research Infervision Medical Technology Co. Ltd Beijing China

**Keywords:** 3D reconstruction, lobectomy, lung cancer, segmentectomy, thoracic surgery

## Abstract

This review presents a comprehensive overview of recent advancements and clinical applications of three‐dimensional (3D) reconstruction technology in thoracic surgery, with a focus on lung cancer surgery. The widespread adoption of chest computed tomography (CT) screening has increased the detection rates of early‐stage lung cancers, facilitating a transition from traditional lobectomy to parenchymal‐sparing sublobar resections, such as segmentectomy, which demand higher anatomical precision. 3D reconstruction technology significantly improves tumor localization, as well as vascular and bronchial visualization, thereby enhancing surgical accuracy and safety. Its key applications encompass preoperative planning, intraoperative navigation, real‐time localization, vascular and airway visualization, and postoperative pulmonary function assessment, collectively contributing to improved surgical outcomes and patient prognosis. Recent innovations in artificial intelligence have streamlined and automated the reconstruction process, leading to reduced operative times and increased accuracy. However, challenges persist, including image quality limitations, algorithm robustness, and limited high‐quality clinical evidence. Future integration with emerging technologies such as virtual reality and augmented reality holds promise for achieving personalized, intelligent thoracic surgical procedures. This review aims to systematically evaluate the clinical value of 3D reconstruction technology and explore its future development directions.

## Graphical abstract

A schematic diagram of the utilization and developement of AI enabled 3D reconstruction in thoracic surgery.

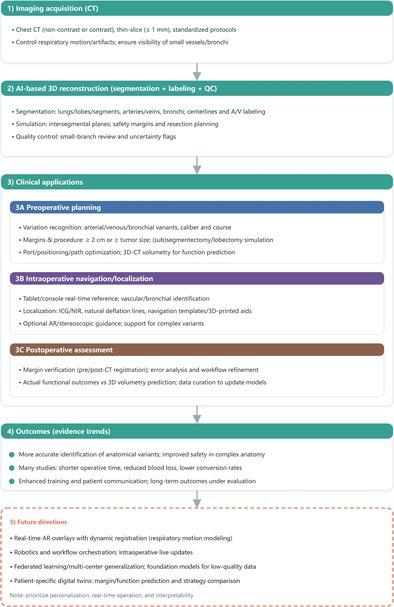

## 1 Introduction

Lung cancer remains the leading cause of cancer‐related mortality worldwide [1]. Surgery provides the best chance for cure in patients with increased CT screening detecting more ground‐glass nodules and small peripheral cancers; traditional surgical approaches are being reconsidered. While lobectomy with lymph node dissection has been the standard for early‐stage NSCLC for three decades, recent major trials (JCOG0802 [2] and CALGB 140503 [3]) demonstrate that sublobar resection, particularly segmentectomy, is equally effective for small, peripheral lesions.

The standardized implementation of sublobar resection imposes higher demands on the precision of identifying pulmonary lesions and adjacent anatomical structures. Traditionally, thoracic surgery has relied primarily on two‐dimensional (2D) computed tomography (CT) imaging for the evaluation of pulmonary anatomy. However, 2D imaging has inherent limitations in accurately depicting complex anatomical variations, which may increase the risk of intraoperative vascular misidentification and inadequate surgical margins [4]. 3D reconstruction technology, first introduced in the 1970s, has gradually evolved and found applications in various surgical disciplines. Compared to conventional 2D CT, 3D reconstructions based on chest CT offer significant advantages in lesion localization, anatomical structure delineation, surgical approach selection, and preoperative planning efficiency. Nevertheless, manual or semi‐automated reconstruction methods remain time‐consuming, thereby limiting their routine clinical application mainly to complex sublobar resections [5].

In recent years, artificial intelligence (AI) has significantly improved both the quality and efficiency of 3D reconstruction processes. AI has facilitated the transition from labor‐intensive manual or semi‐automated segmentation methods to high‐precision, automated segmentation processes, thereby accelerating the integration of 3D reconstruction into routine clinical practice [6].

Currently, the modalities of 3D reconstruction in thoracic surgery have become increasingly diverse, encompassing traditional 2D visualization, interactive navigation through virtual reality (VR) and augmented reality (AR), and physical 3D‐printed anatomical models [7]. However, despite the proliferation of 3D reconstruction algorithms and their clinical applications, a systematic evaluation and synthesis of their efficacy and utility remain lacking. The goal of this review is to provide a comprehensive overview of the technological evolution, recent clinical applications, and outcomes of 3D reconstruction technologies in the field of thoracic surgery.

## 2 The Evolution of 3D Reconstruction Technology in Thoracic Surgery

The advancement of computer vision technology has significantly propelled the innovation and widespread application of 3D reconstruction methods in medical imaging. 3D reconstruction has evolved from traditional techniques such as contour reconstruction and maximum intensity projection (MIP) to more advanced approaches based on convolutional neural networks (CNNs) and point spread functions (PSFs). These improvements in reconstruction efficiency and accuracy have made it feasible to extend the application of 3D reconstruction from complex cases such as segmental lung resections to a broader population of surgical patients.

### 2.1 Classification of 3D Reconstruction Technologies

#### 2.1.1 Consistency‐Based Volume Rendering Technology

This technology represents one of the foundational techniques for 3D reconstruction in medical imaging. It relies on threshold processing and consistency computations applied to image pixels and voxel attributes, such as intensity and opacity, to generate a visual representation that transitions from 2D slices to a 3D model. Prominent algorithms within this category include MIP, Shaded Surface Display (SSD), surface reconstruction, and ray tracing.

As early as 1996, research employed techniques such as MIP and volume rendering to achieve 3D reconstructions of pulmonary lesions and adjacent anatomical structures from CT and MRI data [8]. These efforts aimed to enhance preoperative planning. Initial investigations also utilized SSD and MIP methods for reconstructing pulmonary arteries and tumors in three dimensions, thereby supporting the diagnosis of vascular infiltration [9]. Today, such technologies remain integral to clinical practice, facilitating the rapid reconstruction of blood vessels and bony structures to assist in diagnosis.

However, it is important to note that for applications demanding higher levels of precision—such as surgical planning and navigation—these methods present certain limitations. Firstly, these techniques often depend on empirically determined threshold values; failure to capture tissue details within these thresholds can result in blurred tissue boundaries. Secondly, they do not fully account for the spatial distribution and physical properties of volumetric data. Consequently, the reliance on pixel intensity “consistency” features restricts the geometric accuracy of reconstructed models, especially in complex anatomical regions.

#### 2.1.2 Direct Volume Rendering Technique

The Direct Volume Rendering (DVR) technique explicitly models the optical properties of volumetric data—such as CT or MRI voxels—by simulating the attenuation, scattering, and emission of light as it propagates through the volume. This process yields photorealistic 3D visualizations without the need for explicit surface extraction. Unlike surface‐based or consistency‐based rendering approaches, DVR synthesizes images directly from voxel opacity and color information, thereby retaining the complete volumetric data and facilitating detailed visualization of complex anatomical structures, including soft tissues and intra‐organ features.

The core pipeline of DVR consists of three primary stages: sampling, classification, and compositing. Several algorithms implement this process, including ray casting, texture mapping, splatting, and shear‐warp, all of which have been widely employed in soft tissue visualization and dynamic organ reconstruction tasks. These applications include respiratory motion analysis and tumor motion tracking [10, 11].

It is important to recognize that DVR techniques are computationally demanding, requiring high‐performance hardware to operate efficiently. When processing high‐resolution volumetric datasets, challenges such as substantial memory consumption and increased rendering latency may arise. Nevertheless, recent advancements in computational power and deep learning technologies have opened new avenues for integrating DVR with high‐performance computing and AI. Such integration holds significant promise for enabling real‐time, intelligent 3D reconstructions, which are poised to play a pivotal role in the accurate diagnosis and treatment of complex diseases.

#### 2.1.3 Regularization‐Based Surface Reconstruction Techniques

These methods leverage mathematical modeling and energy function optimization to infer 3D surface structures from 2D image data, incorporating prior knowledge to enhance reconstruction fidelity. By integrating regularization terms into the optimization process, these techniques effectively mitigate model overfitting and balance the fidelity to data with the enforcement of prior constraints, thereby improving both the accuracy and stability of the reconstructed surfaces. Numerous algorithms have been developed within this framework, including approaches that model the relationship between volumetric data and 2D slices using the PSF matrix, as well as iterative methods designed to correct motion artifacts [12]. Hierarchical Deformable Models (HDM) have been successfully applied to spinal reconstruction through a three‐step process involving landmark detection, global registration, and local orientation, while utilizing Conditional Random Field Models (CRBM) to extract multimodal image features for the construction of high‐precision spinal models [13].

This class of techniques employs regularization constraints and data‐driven strategies, reducing subjective errors common in traditional thresholding methods. Consequently, they are suitable for reconstructing complex anatomical structures. When integrated with deep learning frameworks, these methods can enable end‐to‐end reconstruction workflows, significantly reducing the need for manual intervention. However, the success and robustness of these approaches heavily depend on the accuracy and comprehensiveness of the prior knowledge employed. Limitations in the diversity of anatomical samples can impair the generalizability of the resulting models. A major challenge faced by these techniques is the substantial annotation effort required for large volumes of medical data, which can be resource‐intensive and time‐consuming.

### 2.2 Commonly Used 3D Reconstruction Software

Currently, the 3D reconstruction software commonly used in clinical pulmonary surgery can be categorized into open‐source and closed‐source systems (Table 1). Open‐source platforms such as Horos and Fiji offer accessible and user‐friendly environments for fundamental 3D reconstruction tasks. These systems allow clinicians to actively participate in the reconstruction process, providing direct control and manipulation of the imaging data. However, when it comes to advanced procedures—such as high‐precision vascular segmentation or the detailed reconstruction of bronchial trees, these open‐source solutions often demand increased manual input and extended processing time. Furthermore, the quality and efficiency of reconstructions heavily depend on the operator's experience and skill in executing specific steps, which can introduce variability [14]. In contrast, closed‐source reconstruction systems, such as Synapse Vincent, MIMICS, and InferOperate Thorax, are more prevalent in clinical practice due to their automation capabilities and integrated functionalities. These systems offer comprehensive solutions, particularly suited for complex surgical planning. For instance, they can automatically simulate intersegmental planes based on bronchial anatomical features [15], and perform highly accurate, automated segmentation of pulmonary segments [16]. Their streamlined workflows contribute to increased consistency, reduced manual workload, and improved reliability in clinical settings.

**TABLE 1:** Commonly used 3D reconstruction software systems in thoracic surgery.

Software system	Regulatory approval	Underlying principle	Reconstruction mode and efficiency
Synapse Vincent	Closed‐source: FDA, NMPA, CE	Traditional algorithms; AI integrated in newer versions	Fully automatic
Visible Patient	Closed‐source: FDA, CE	Traditional algorithms	Semi‐automatic
InferOperate Thorax	Closed‐source: NMPA	Deep learning based on convolutional neural networks (CNNs)	Fully automatic, ~5 min
Ceevra Reveal 3	Closed‐source: FDA	Machine learning and computer vision algorithms	Cloud‐based, within 2 days
Ziostation 2	Closed‐source: FDA	Unknown	Semi‐automatic
REVORAS	Closed‐source: CE	AI‐integrated functionality	Fully automatic, within minutes
Materialize Mimics Innovation Suite 23	Closed‐source: FDA, CE, NMPA	Traditional algorithms	Semi‐automatic
IQQA‐3D	Closed‐source: NMPA, FDA	Traditional algorithms	Semi‐automatic
Deepinsight Platform	Closed‐source: Not specified	Traditional algorithms	Semi‐automatic
ExoView	Closed‐source: Not specified	Traditional algorithms	Semi‐automatic
Horos Project/OsiriX	Open‐source software	Traditional algorithms	Semi‐automatic
Fiji (ImageJ)	Open‐source software	Traditional algorithms	Semi‐automatic
3D Slicer	Open‐source software	Traditional algorithms	Semi‐automatic

Abbreviations: CE, conformité européenne; FDA, food and drug administration; NMPA, national medical products administration.

Currently, open‐source systems still exhibit limitations in terms of clinical applicability and the depth of validation (Tables 2 and 3). Consequently, proprietary (closed source) systems continue to dominate in routine medical practice. Notably, 3D reconstruction systems applied in pulmonary surgery are primarily evaluated based on the following performance aspects:

**TABLE 2:** Evaluation of clinical effectiveness.

Study	Application scenario	Study design	Comparison groups	Sample size (3D/control)	Primary outcome(s)	Result (3D vs. control)	*p*
Chen et al. (2025) [4]	Preoperative planning	Retrospective	3D‐CT vs. 2D‐CT	140	Accuracy of anatomical variation identification	+8% accuracy (41% fewer errors)	*p* < 0.01
Cui et al. (2020) [17]	Preoperative planning	Retrospective	3D‐CT vs. 2D‐CT	52	Accuracy in identifying segmental pulmonary arteries	95.7% vs. 100%	*p* = 0.013
Li et al. (2025) [18]	Preoperative planning	Prospective	3D‐CT vs. 2D‐CT (by junior and experienced surgeons)	49	Surgical planning success rate	Improved in all surgeons: Junior A: 40.8%→87.8% Junior B: 49.0%→89.8% Experienced C: 83.7%→100% Experienced D: 75.5%→95.9%	*p* < 0.0125
Chu et al. (2021) [19]	Preoperative planning	Retrospective	Single‐arm cohort	26	Localization success rate	96.2%	—
Chen et al. (2020) [20]	Preoperative planning	Retrospective	3D‐CT + 3D printed model vs. 3D‐CT only	51/38	Surgical approach conversion Surgical method conversion	0% vs. 10.5% 0% vs. 10.5%	*p* = 0.03 *p* < 0.03
Hojski et al. (2025) [21]	Preoperative planning	Retrospective	3D‐CT vs. 2D‐CT	37/63	Complex surgery rate Thoracotomy conversion rate	89% vs. 38% 0% vs. 19%	*p* < 0.001 *p* = 0.037
Wang et al. (2022) [22]	Preoperative planning	Retrospective	3D‐CT vs. 2D‐CT	45/55	Operative time Air leak rate (post‐op days 1–3)	111.4 vs. 127.1 min 11.9% vs. 30.9%	*p* = 0.007 *p* = 0.027
Xue et al. (2018) [23]	Preoperative planning	Retrospective	3D‐CT vs. 2D‐CT	36/32	Operative time Margin adequacy	111 vs. 139 min 0 vs. 4 cases	*p* = 0.03 *p* = 0.04
Qiu et al. (2020) [24]	Preoperative planning	Retrospective	3D‐CT vs. 3D Model vs. non‐3D‐CT	131/31/136	Operative time Intraoperative blood loss	99.6 vs. 116.1 vs. 125.1 min 12.9 vs. 20.9 vs. 18.2 mL	—
He et al. (2024) [25]	Preoperative planning	Retrospective	3D‐CT vs. 2D‐CT	148/117	Intraoperative blood loss	77.5 ± 29.0 mL vs. 120 ± 68.5 mL	*p* < 0.001

**TABLE 3:** Algorithm performance evaluation.

Study	Segmentation target	Algorithm/method	Evaluation metrics	Performance results
Chen et al. (2022) [5]	Pulmonary vessels and bronchi (non‐contrast CT)	Fully automated DL‐based reconstruction	Detection accuracy; Classification accuracy	Detection: 85%; Classification: 80%
Chen et al. (2022) [26]	Segmental arteries and lobar veins	U‐Net	Case‐level and structure‐level accuracy	Case‐level overall: 82.8%; Arteries: 79.7%; Veins: 96.3%
Cui et al. (2020) [17]	Segmental pulmonary arteries	3D reconstruction software (Exoview)	Detection accuracy	Exoview: 95.7% (132/138); Thin‐slice MDCT: 100%
Yao et al. (2017) [14]	Pulmonary vessels and bronchi	OsiriX (volume rendering, open‐source)	Feasibility and practicality	Clear anatomical display; helpful for preoperative planning and variation identification
Yang et al. (2016) [16]	Segments, vessels, bronchi, nodules	LOTIS 3D imaging system	Reconstruction quality (arterial levels); Feasibility for localization/planning	Reconstructed up to 5th‐level arteries; clear anatomical adjacency
Nakazawa et al. (2021) [27]	Pulmonary vessels and bronchi (non‐contrast CT)	Ziostation software	Technical feasibility; Comparison with contrast‐enhanced CT	Similar quality to enhanced CT; occasionally better for small vessels
Lin et al. (2024) [28]	Lung, tumors, vascular tree	Vessel segmentation, subvascular tree matching, image registration	Target registration error (TRE)	TRE < 2.5 mm for all cases

#### 2.2.1 Reconstruction Accuracy and Efficiency

The accuracy of 3D reconstruction is critical for understanding the spatial relationships between lesions and adjacent structures, influencing surgical decisions. Accurate reconstruction enables effective intervention and precise lesion resection. Cui et al. [17] assessed the accuracy of their ExoView software, which identified 95.7% of segmental pulmonary arteries on 3D images compared to intraoperative findings, with a margin of error of less than 1.4 mm for the six missed arteries. Similarly, Chen et al. [5] introduced a fully automated 3D pulmonary reconstruction system using deep learning and CNNs, demonstrating an 85% detection accuracy and 80% classification accuracy. Additionally, their use of U‐Net architecture for segmental artery and lobar vein segmentation resulted in an overall accuracy of 82.8%, with 79.7% for segmental arteries and 96.3% for lobar veins [26].

Tokuno et al. [29] developed the Resection Process Map (RPM), a 3D reconstruction system that, using STL format and auxiliary software, achieved 98.6% accuracy in detecting vascular branches and successfully identifying all bronchial structures.

The efficiency of 3D reconstruction significantly impacts its clinical application, as traditional methods can be labor‐intensive and time‐consuming, limiting their use to complex cases. Advances in AI‐powered reconstruction have notably improved efficiency. Chen et al. demonstrated that deep learning‐based algorithms reduced reconstruction times to around 280 s, compared to 30 min for traditional manual methods using Mimics software [4, 26]. Automated software, like the Synapse Vincent system, further enhances efficiency by producing high‐quality, patient‐specific 3D models within minutes. This integration of automation and software improves both speed and accuracy, facilitating preoperative planning, simplifying complex surgeries, and enhancing intraoperative guidance, ultimately improving surgical outcomes and efficiency [30, 31].

#### 2.2.2 Generalization Capability

Regarding the generalization of 3D reconstruction methods, previous approaches have predominantly depended on contrast‐enhanced CT imaging. Nonetheless, recent advancements in algorithmic optimization have markedly improved the processing efficiency of non‐contrast CT scans. Nakazawa et al. demonstrated that a 3D reconstruction technique utilizing non‐contrast CT data can accurately model the pulmonary vasculature. This methodology initially exploits the differences in CT attenuation between lung parenchyma and soft tissue to perform an initial segmentation of the pulmonary vessel network. Subsequently, specific anatomic landmarks in the hilum—such as the main pulmonary artery trunk and the left atrium—are tracked, and targeted inclusion and exclusion operations are applied to delineate and separate arterial and venous structures with high precision. For finer peripheral vasculature, the software's “extension” function facilitates automatic tracking and reconstruction of small vessels. Studies have shown that the quality of 3D vascular images reconstructed from non‐contrast CT using this approach is comparable to, or in some cases exceeds, that obtained from contrast‐enhanced scans—particularly in detecting small vessels—due to meticulous manual adjustments incorporated during processing [27]. Furthermore, Chen et al. confirmed that AI significantly enhances both the efficiency and accuracy of 3D reconstructions on non‐contrast CT images. Additionally, Fukuta et al. conducted comparative validation of the Synapse Vincent AI system on contrast‐enhanced and non‐contrast CT images. The system achieved recognition rates for pulmonary artery branches of 98.9% with contrast‐enhanced scans and 96.6% with non‐contrast scans; for pulmonary vein branches, recognition rates were 85.7% and 82.1%, respectively [32]. These findings demonstrate that high‐quality reconstruction is feasible without the use of contrast agents, highlighting significant progress in AI‐assisted pulmonary vascular modeling. These technological advances effectively mitigate the challenge posed by low contrast in non‐contrast scans, enabling rapid and precise 3D modeling. Such improvements increase the robustness and expand the potential clinical applications of these algorithms [4, 5].

## 3 Clinical Applications of 3D Reconstruction Technology in Thoracic Surgery

**FIGURE 1:**
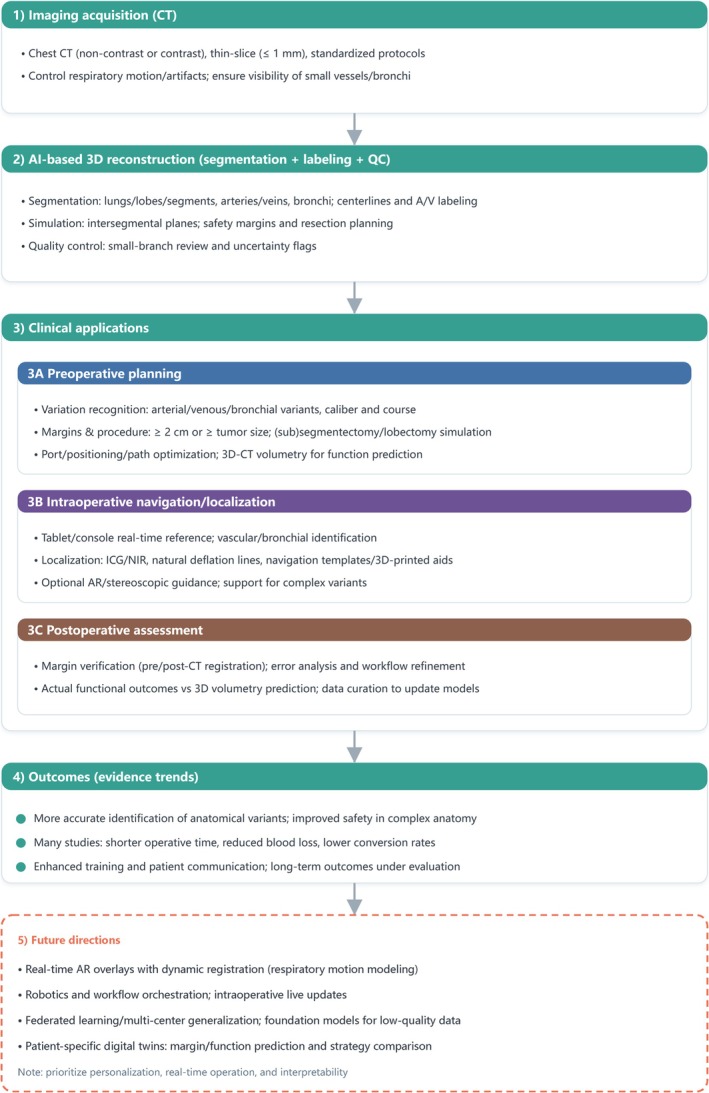
Clinical workflow of AI‐enabled 3D reconstruction in thoracic surgery.

Utilizing its ability to generate detailed 3D reconstructions from CT imaging data, 3D reconstruction technology has become integral to multiple critical stages of thoracic surgery. Its applications encompass preoperative planning, intraoperative lesion localization, postoperative pulmonary function assessment, and educational and training purposes. A considerable amount of research has demonstrated that the use of 3D reconstruction as an adjunct throughout these surgical phases significantly enhances perioperative outcomes (Figure 1). This part of the review aims to explore the various clinical scenarios where 3D reconstruction is applied and to evaluate its impact on surgical efficacy and patient safety.

### 3.1 Application in Doctor‐Patient Communication

Due to the highly specialized nature of medicine, a natural cognitive gap often exists between physicians and patients, which can hinder effective communication to some extent. In the context of thoracic surgery, particularly when obtaining informed consent for thoracoscopic lobectomy, it is crucial that patients and their families comprehend details such as tumor location and size, surgical approach, and potential postoperative complications. Relying solely on textual explanations or verbal descriptions can make it difficult for patients and their families to intuitively grasp complex information about their condition and treatment options.

3D reconstruction technology provides a tangible and effective tool for enhancing doctor‐patient communication, especially when combined with 3D printing technology. Yoon et al. [33] conducted a survey involving patients with early‐stage lung cancer, demonstrating that the use of 3D‐printed models during informed consent significantly enhanced patients' understanding of their condition. Additionally, a study by Qiu et al. [24] exploring the use of 3D‐printed models as surgical aids found that 88% of surgeons believed these models enhanced communication with patients and their families. Moreover, patients and their relatives found 3D‐printed models to be more engaging and easier to understand than virtual 3D models. Overall, 3D reconstruction technology has the potential to bridge comprehension gaps in doctor‐patient interactions, thereby promoting more harmonious and effective relationships.

### 3.2 Application in Preoperative Planning

Accurate assessment of anatomical structures is a crucial component of preoperative planning, as it ensures both surgical safety and the adequacy of resection margins. 3D reconstruction significantly enhances thoracic surgeons' understanding of complex pulmonary anatomy by transforming conventional 2D CT images into more intuitive and comprehensible visual representations. This technological advancement allows for precise identification of anatomical variations, accurate determination of resection margins, improved localization of nodules, optimized planning of surgical approaches, and more reliable prediction of postoperative pulmonary function.

#### 3.2.1 Accurate Identification of Anatomical Variations

Studies have demonstrated that the incidence of anatomical variations in human pulmonary vessels exceeds 30% [34, 35]. The accuracy of thoracic surgeons in identifying these variations using 2D‐CT alone is less than 80% [26, 36]. In contrast, 3D reconstruction significantly enhances detection accuracy, achieving rates between 85% and 87% [4, 5]. Laven et al. [36] confirmed that preoperative 3D reconstruction can identify up to 80% of anatomical variations that are often undetectable on 2D imaging. These subtle variations, frequently overlooked in conventional imaging, are a major contributing factor to inadvertent intraoperative vascular injuries [25, 37].

Moreover, Petrella et al. reported that AR technology, implemented via holograms, demonstrates superior accuracy in preoperative assessment of pulmonary vascular anatomy compared to standard CT images. Specifically, hologram‐assisted assessments yielded an inter‐observer agreement of 98% regarding the number of lobar arteries—significantly higher than the 85% observed with CT imaging. In the upper lobe region, which is characterized by a higher prevalence of anatomical variations, the mean difference between the number of arteries identified via holograms and intraoperative findings was 0.17 ± 0.46, markedly better than the 0.67 ± 1.08 discrepancy seen with CT images (*p* = 0.029). These findings suggest that AR technology enables more precise identification of anatomical structures, thereby facilitating more accurate surgical planning [38].

A thorough understanding of the origin, course, and variation of pulmonary vessels prior to surgery enables surgeons to optimize surgical approaches, anticipate anatomical challenges, and effectively avoid injury to vital vessels. This preoperative precision can reduce intraoperative blood loss, decrease the incidence of related complications, and ultimately enhance surgical safety [20, 39].

Furthermore, Chen et al. demonstrated that AI‐based 3D reconstruction systems can improve the accuracy of identifying anatomical variations. In a multicenter, multi‐reader, multi‐case study involving 140 chest CT scans and 10 physicians with varying levels of experience, AI‐assisted 3D reconstruction increased the identification rate of pulmonary anatomical structures by 8% and decreased error rates by 41%, compared to conventional 2D CT guidance. These improvements were consistent across all participating surgeons, providing robust evidence supporting the integration of AI‐enhanced 3D reconstruction into pulmonary surgical planning [4].

Overall, these advances in imaging and technology significantly contribute to safer, more precise pulmonary surgeries by enabling better preoperative anatomical assessment and reducing the risk of intraoperative complications.

#### 3.2.2 Accurate Assessment of Resection Margins

According to the guidelines established by the National Comprehensive Cancer Network (NCCN) [40] and the Clinical Practice Guidelines for Lung Cancer by the Chinese Medical Association [41], a minimum surgical margin of 2 cm is recommended for pulmonary nodules. Nevertheless, despite the high resolution of 2D CT imaging in sagittal and coronal planes, the vertical (cranio‐caudal) dimension is frequently underappreciated, potentially leading to incomplete resection margins. Traditional 2D CT imaging heavily depends on the surgeon's experience for planning surgical boundaries, which may result in suboptimal resections, especially when performed by less experienced surgeons. He et al. [25] reported a case in which exclusive reliance on 2D CT led to inadvertent pulmonary artery injury, necessitating an extended resection. A comparative study further highlighted the advantages of 3D reconstruction, demonstrating that patients whose surgical planning employed 3D imaging (*n* = 36) achieved a 100% rate of satisfactory surgical margins. In contrast, in a control group using only 2D CT (*n* = 32), four patients (4/32) required additional wedge resections due to inadequate margins—a difference that was statistically significant [23]. Currently, postoperative margin assessment remains largely dependent on subjective evaluation, lacking standardized, objective, quantitative criteria. To address this limitation, semi‐automated systems have been developed to facilitate postoperative margin analysis. These systems utilize algorithms such as Thin Plate Spline (TPS) registration to align preoperative virtual margins with the actual postoperative specimen, enabling quantitative assessment of margin discrepancies, which average approximately 2.8 ± 1.3 mm [28]. This technological advance provides an objective framework for evaluating the radicality of lung cancer surgeries. Further investigation by Chen et al. analyzed errors in resection margin planning and confirmed that 3D reconstruction significantly reduced the incidence of inadequate margins. This improvement has contributed to more precise sublobar resections, helping surgeons avoid insufficient margins [4].

The benefits of 3D imaging have also been validated at the histopathological level, with the technology playing a crucial role in ensuring accurate tumor excision, increasing the rate of negative margins, and ultimately enhancing patient outcomes. Nakamoto et al. employed 3D‐CT angiography to guide super‐selective segmentectomy, enabling the planning of margins exceeding the tumor diameter or ≥ 20 mm in patients with deep pulmonary nodules. All patients in this cohort achieved pathologically negative margins, and among those who underwent segmentectomy alone for early‐stage lung cancer, 5‐year overall survival and disease‐free survival rates were both 100%. Notably, no local recurrences were observed during follow‐up [42]. Similarly, Kanzaki et al. performed thoracoscopic multi‐subsegmental resections for non‐small cell lung cancer (NSCLC), utilizing patient‐specific virtual 3D pulmonary models. Preoperative simulation ensured adequate surgical margins (> 2 cm) in all cases, with no instances of local or margin recurrence during follow‐up—despite some occurrences of distant metastasis [43]. Accurate identification of intersegmental planes is also vital in segmentectomy. Xu et al. first simulated intersegmental planes using 3D reconstruction based on bronchial branching patterns. These virtual planes were later validated against the anatomical locations of intersegmental veins, with the pulmonary artery serving as a key anatomical landmark. This approach proved highly accurate in delineating intersegmental planes, offering strong objectivity and reproducibility. Such precision aids surgeons in planning resection boundaries, ensuring complete (R0) resection of early‐stage lung lesions while maintaining adequate safety margins [15].

### 3.3 Intraoperative Lesion Localization, Structure Identification, and Navigation

In contrast to traditional 2D imaging modalities, 3D reconstruction techniques provide a significantly more intuitive visualization of lesions and their surrounding anatomical structures. This improved spatial perception facilitates more accurate intraoperative assessment and decision‐making by surgeons. An increasing volume of literature consistently highlights the invaluable intraoperative benefits of 3D reconstruction, often incorporating advanced technologies such as 3D printing and polarized 3D glasses to further enhance surgical guidance and situational awareness.

#### 3.3.1 Intraoperative Target Nodule Localization

Locating pulmonary nodules during wedge resection remains a considerable clinical challenge. Traditional localization techniques often involve invasive physical marking methods, which can be associated with procedural risks and technical limitations. In contrast, 3D reconstruction technology offers a novel, noninvasive approach to improve localization accuracy. Building on meticulous surgical simulation and preoperative planning based on 3D reconstructed CT images, Chu et al. proposed an innovative strategy for pulmonary nodule localization. This method involves temporarily occluding the pulmonary artery supplying the target lesion during surgery, in conjunction with intravenous administration of the fluorescent dye indocyanine green (ICG). Utilizing near‐infrared (NIR) imaging, the perfusion boundaries of the targeted segment are clearly delineated on the pleural surface, providing precise guidance for subsequent wedge resection. Clinical validation demonstrated the effectiveness of this approach: out of 26 patients enrolled, successful localization and resection were achieved in 25 cases [19]. Similarly, other research groups have reported promising findings. For instance, Zhao et al. utilized a target segmental planning approach based on 3D reconstruction combined with a noninvasive intraoperative lung deflation technique to localize GGNs. This method achieved successful resection with a mean localization time of 6.9 min and a localization accuracy rate of 90.6% [44]. Additionally, Zhao et al. integrated 3D reconstruction with a compass‐guided localization strategy, allowing direct, CT‐free lesion localization within the operating room. The distance from the nodule to the pleural surface marker ranged from 0 to 25 mm (mean: 8.0 ± 3.9 mm), with a high localization accuracy of 97.6% (81 out of 83 lesions) [45]. Moreover, 3D printing technology has demonstrated significant potential in pulmonary nodule localization. Several studies have shown that patient‐specific navigation templates produced via 3D printing can accurately guide nodule localization, thereby facilitating intraoperative identification and resection [46, 47].

#### 3.3.2 Intraoperative Anatomical Structure Identification and Navigation

Numerous studies have demonstrated that the visualization of reconstructed 3D images on tablet computers within the operating room significantly enhances the identification of critical anatomical structures and their intricate spatial relationships. The capacity to interactively view and manipulate these images intraoperatively markedly improves the surgeon's understanding of surgical anatomy, thereby augmenting their ability to perform anatomical resections safely and accurately [48, 49]. Furthermore, Chen et al. reported that the utilization of patient‐specific 3D‐printed lung models effectively aids surgeons in intraoperative recognition of relevant surgical landmarks. These models facilitate easier localization of target lesions, allow for the selection of pivotal anatomical points as reference markers, and enable the identification of anatomical variations from multiple perspectives throughout the procedure [20]. In addition, Kanzaki et al. explored the application of 3D polarized glasses for binocular stereoscopic navigation. This technique permits real‐time display of 3D reconstructed images, serving as a valuable intraoperative navigation aid during video‐assisted thoracoscopic surgery (VATS). Their findings indicated that this approach not only simplified and improved the accuracy of identifying bronchovascular structures but also contributed to achieving optimal surgical resection margins [50].

#### 3.3.3 Surgical Approach Planning

Le et al. demonstrated that preoperative application of three‐dimensional (3D) reconstruction technology can enhance surgeon confidence through more effective surgical planning and intraoperative usage. Real‐time visualization of 3D models during the procedure assists surgeons in gaining a more comprehensive understanding of the patient's anatomical structures, particularly anatomical variations. Additionally, this approach facilitates optimization of patient positioning and puncture site design, thereby improving surgical workflow and potentially increasing procedural safety [51].

#### 3.3.4 Postoperative Pulmonary Function Prediction

Accurate prediction of postoperative pulmonary function is crucial for effective surgical risk assessment and optimizing patient outcomes following pulmonary resection. Traditional predictive methods, such as the segment counting approach, are inherently limited, as their accuracy can be significantly affected by factors like the location of the resected lobe. In recent years, 3D‐CT volumetry has emerged as a more precise tool for estimating postoperative lung function. Shibazaki et al. demonstrated that the accuracy of 3D‐CT volumetry in predicting forced expiratory volume in 1 s (FEV_1_) after various lobectomies was independent of the location of the resected lobe (*p* = 0.370), indicating superior consistency compared to the segment counting method. Conversely, the segment counting approach exhibited significant variability in predictive accuracy depending on the resected lobe (*p* < 0.001), often overestimating lower lobe volumes and underestimating upper lobe volumes. This highlights the advantage of 3D‐CT volumetry in providing a quantitative assessment based on actual anatomical data, enabling precise calculation of the volume of both removed and residual lung tissue, including parenchyma, vasculature, bronchi, and lesions. Such detailed assessment allows for more individualized and accurate prediction of postoperative lung function [52]. Furthermore, Kang et al. [53] compared CT volumetry with perfusion scans for predicting postoperative pulmonary function, concluding that CT volumetry is a valid and reliable alternative. However, the accuracy of 3D‐CT volumetry can still be influenced by other factors. Existing literature indicates that the number of stapler firings used during interlobar fissure management and the presence of interstitial pneumonia are significant factors affecting its predictive precision (*p* < 0.001) [52]. In summary, 3D‐CT volumetry significantly improves the accuracy and reliability of postoperative pulmonary function prediction by providing individualized, anatomy‐based quantitative assessments that are independent of the resected lobe's position. This technological advancement offers a more robust foundation for clinical decision‐making and tailored patient management strategies.

#### 3.3.5 Application of 3D Reconstruction in Surgical Education and Training

Liu et al. conducted a pivotal study investigating the utility of 3D reconstruction in assisting less experienced surgeons undertaking thoracoscopic anatomical partial lobectomy. Their findings revealed that thoracoscopic anatomical lobectomy guided by interactive quantitative 3D reconstruction planning could be proficiently mastered by surgeons after completing only 30 cases. This demonstrated a significantly accelerated learning curve inflection point (30 cases) [54] when compared to previously reported benchmarks for thoracoscopic anatomical segmentectomy (84 cases) [55]. These compelling findings underscore the substantial potential of 3D reconstruction technology to enhance surgical training programs and support the professional development of young surgeons.

#### 3.3.6 Clinical Efficacy of 3D Reconstruction Technology in Lung Surgery

Numerous comparative studies have consistently demonstrated that preoperative planning based on 3D reconstruction enhances the accuracy of resectability assessment and facilitates the identification of anatomical variations that could potentially lead to intraoperative complications. This improvement in anatomical visualization subsequently translates into better perioperative outcomes. Concerning operative time, most investigations indicate that 3D reconstruction effectively reduces surgical duration, particularly in complex segmentectomies [22, 23, 24, 56]. This efficiency primarily results from the clear visualization of anatomical structures and precise surgical pathway planning provided preoperatively, which collectively minimizes intraoperative exploration and decision‐making time. Furthermore, the precise navigation enabled by 3D reconstruction generally contributes to a reduction in intraoperative blood loss. Multiple studies report significant decreases in blood loss within the 3D‐assisted group, attributed to the accurate preoperative identification and simulation of vascular structures [20, 23, 24, 25], thereby enhancing surgical safety. Although some investigations did not find statistically significant differences, a consistent trend toward reduced bleeding was observed across these studies [22, 23]. In addition, 3D reconstruction demonstrates a clear advantage in lowering the rate of conversion to thoracotomy and in reducing the need for unplanned surgical modifications. Chen et al. and Hojski et al. [20, 21] both report significantly lower conversion rates in the groups utilizing 3D planning compared to control groups. Regarding postoperative complications, several studies have documented a positive impact of 3D reconstruction, with notable reductions in the overall incidence and severity of complications, including those classified as Clavien‐Dindo Grade III/IV [21, 25, 56]. However, some research, such as studies by Xue et al. and Chen et al. [20, 23], did not observe statistically significant differences in overall complication rates between groups, which could be attributable to variations in sample size, surgical procedures, and the definitions of complications across different studies. In summary, 3D reconstruction technology offers significant advantages in preoperative planning, accurate anatomical visualization, and intraoperative guidance, all of which contribute to enhanced surgical safety. The preponderance of evidence supports its utility in shortening operative times, minimizing intraoperative blood loss, reducing conversion rates to thoracotomy, and potentially decreasing certain postoperative complications, ultimately improving perioperative patient outcomes. While the findings across studies are not entirely uniform for every outcome measure, the wide recognition of its potential in managing complex anatomical scenarios and augmenting surgical precision underscores its growing relevance in thoracic surgery.

## 4 Challenges, Limitations, and Future Directions of 3D Reconstruction Technology in Pulmonary Surgery Applications

Despite the significant potential demonstrated by 3D reconstruction technology in the field of pulmonary surgery, it still confronts a series of challenges, limitations, and unresolved controversies at both the technical and clinical practice levels.

### 4.1 Impact of Image Quality on Reconstruction Accuracy

The foundational prerequisite for accurate 3D reconstruction is the intrinsic quality of the CT images. Chen‐Yoshikawa and Date emphasized that the quality of source images and the efficiency of their acquisition constitute critical bottlenecks limiting the widespread adoption of this technology. They underscored that high‐quality 3D‐CT images are essential for precise preoperative simulations [31].

Research by Lin et al. further highlights how variations in image quality significantly affect key steps within the reconstruction workflow. They observed that factors such as different scanning equipment, variations in acquisition parameters, and fluctuating patient respiratory phases can all influence the accuracy and consistency of vascular tree segmentation. This variability may impede the precise matching of anatomical features, potentially resulting in inadequate preoperative vascular delineation and unsuccessful registration of vascular structures when compared to postoperative images. As a consequence, the overall accuracy of image registration is compromised [28]. This indicates that CT datasets exhibiting issues such as inconsistent scanning parameters, significant noise, or severe respiratory motion artifacts may surpass the handling capacity of current reconstruction algorithms, leading to diminished accuracy or even registration failure. Similarly, if the images lack sufficient anatomical detail or if vasculature is poorly visualized, the potential accuracy of the reconstruction process is inherently limited. Addressing these challenges requires a comprehensive, multi‐faceted strategy. Firstly, efforts should be directed toward standardizing and optimizing imaging protocols to improve data quality at the source. Secondly, the development of advanced image preprocessing and enhancement algorithms is critical to elevate the quality of input data. Thirdly, it is imperative to innovate more robust 3D reconstruction and segmentation algorithms capable of handling lower‐quality inputs. Additionally, establishing large‐scale, diverse benchmark datasets—including low‐quality images—is essential for the validation and improvement of these algorithms. Collectively, these research initiatives will be pivotal in advancing the reliability, accuracy, and clinical applicability of 3D reconstruction technology, particularly in complex and challenging clinical scenarios.

### 4.2 Limitations in Scope of Application and Reconstruction Accuracy

Several studies have highlighted that the current clinical validation of 3D reconstruction techniques offers limited coverage of rare anatomical variations. As a result, there remains a notable lack of supporting data within patient subgroups where 3D reconstruction could potentially yield the greatest clinical benefits [4, 18]. To overcome this limitation, the development of dedicated, population‐specific datasets is essential, facilitating more robust evaluation and validation of these technologies across diverse anatomical presentations. Currently, there is a significant gap in 3D reconstruction algorithms tailored specifically for postoperative CT imaging. Patients undergoing re‐operative procedures often exhibit complex anatomical alterations, making reconstruction accuracy particularly challenging. Addressing this gap is a pressing priority, as reliable postoperative models are crucial for surgical planning and intraoperative navigation in these complex cases.

Furthermore, although automated segmentation algorithms, especially those utilizing deep learning approaches, have made substantial progress, robustness remains a critical concern [4]. Existing algorithms can still produce errors or incomplete segmentations, particularly when trying to identify and delineate distal small vessels or structures exhibiting anatomical variations. The performance of these algorithms varies considerably depending on the dataset and the degree of anatomical complexity encountered. As of now, a standardized, highly efficient, and accurate segmentation methodology that performs reliably across different clinical scenarios is lacking [26].

A comparative study demonstrated that, compared to interpretations by experienced radiologists using thin‐slice multidetector CT (MDCT), 3D reconstructions failed to identify all small segmental pulmonary artery branches, with a statistically significant difference noted in the detection rate of these fine branches (*p* = 0.013) [17]. This finding underscores that, while 3D reconstruction represents a valuable adjunctive tool, surgeons must continue to meticulously review the original source images preoperatively. Vigilance is essential to identify potential errors or omissions in the reconstructed models, which may originate from technical limitations or human factors, thereby reducing the risk of intraoperative complications attributable to inaccuracies in the models.

### 4.3 Deficiency in High‐Level Evidence Supporting Clinical Value

Although retrospective studies have indicated that 3D reconstruction technology offers certain benefits—such as reduced operative time, decreased intraoperative blood loss, and lower complication rates—the current high‐quality evidence remains limited [20, 22, 24, 25]. Notably, Chen et al. found that, compared to standard CT, preoperative 3D‐CT did not confer significant advantages in shortening operative time or improving perioperative outcomes during thoracoscopic segmentectomy. The authors partially attributed this “negative finding” to a high baseline performance within their study cohort: the median operative time was already considerably shorter than most reported values in the literature. This likely reflects a higher proportion of straightforward segmentectomies and the extensive surgical experience of the participating surgeons, which may have masked or diminished the potential benefits of 3D‐CT. Additionally, the study might have been underpowered, as the actual operative times were substantially lower than initially anticipated [57].

More broadly, these findings highlight a fundamental limitation of traditional randomized controlled trial (RCT) designs when evaluating advanced medical technologies, especially cognitive support tools such as AI‐assisted 3D reconstruction. Conventional RCT methodologies often struggle to adequately capture the complex interactions between clinicians and novel technologies, including learning curves and variability in user experience—factors that substantially influence real‐world effectiveness. When a technology's primary value lies in aiding complex decision‐making, managing difficult clinical scenarios, or facilitating skill acquisition among trainees, RCTs involving experienced surgeons performing routine procedures may fail to fully assess its potential benefits.

Therefore, interpreting negative or inconclusive RCT results requires critical reflection not only on study design elements such as sample size, patient selection, and endpoint definitions but also on the fundamental limitations of RCTs in evaluating human‐in‐the‐loop healthcare innovations. Future research should consider more sophisticated methodologies—such as stratified analyses, formal learning curve assessments, or adaptive trial designs—to better elucidate the true clinical value of these interventions.

A multicenter retrospective analysis by Chen et al. [4] confirmed that, while 3D reconstruction improves the accuracy of identifying anatomic variations, this improvement has yet to be definitively linked to superior surgical outcomes, such as reduced intraoperative blood loss, shorter operative times, or lower complication rates. Evidently, more high‐level evidence is essential to establish the tangible benefits of this technology on clinical practice, particularly regarding its impact on long‐term patient survival.

Achieving this may require large‐scale studies with ample statistical power to evaluate the influence of optimized workflow components on surgical safety and patient prognoses. Consequently, current research efforts might be better directed toward implementing integrated, end‐to‐end intelligent workflow solutions. Such comprehensive approaches are more likely to produce a substantial, practical impact on overall patient outcomes, ultimately justifying widespread adoption and further development of these advanced technological platforms.

## 5 Conclusion

3D reconstruction technology significantly enhances surgical planning accuracy and intraoperative safety by providing detailed visualization of pulmonary anatomy, particularly complex vascular and bronchial variations. This advanced imaging modality effectively addresses the limitations of traditional 2D imaging, reducing risks such as vascular misidentification and inadequate resection margins, thereby improving short‐term patient recovery outcomes. However, several challenges remain, including increasing reconstruction efficiency, achieving real‐time alignment of static 3D images with the dynamic views encountered during thoracoscopic procedures, and improving the accuracy of microstructural reconstructions. Additionally, evidence supporting long‐term prognostic benefits of this technology is still emerging, representing an ongoing area of research. Future integration of AI promises to further enhance the intelligence and efficiency of 3D reconstruction. Advances in real‐time dynamic navigation combined with technologies like AR are expected to drive thoracic surgery toward higher levels of personalization and precision, ultimately offering patients more tailored therapeutic options, and ultimately offering patients more optimal therapeutic options.

## Author Contributions

**Guihu Lin:** writing – original draft. **Ruzhen Li:** writing – original draft. **Xiao Li:** writing – review and editing. **Dawei Wang:** writing – review and editing. **Xiuyuan Chen:** writing – review and editing.

## Ethics Statement

The authors have nothing to report.

## Conflicts of Interest

Dawei Wang is a paid employee of Infervision Medical Technology Co. Ltd.

## Data Availability

This study did not generate any new datasets. All data analyzed are from publicly available sources, as cited in the manuscript.
